# Potential of Chinese Yam (*Dioscorea polystachya* Turczaninow) By-Product as a Feed Additive in Largemouth Bass (*Micropterus salmoides*): Turning Waste into Valuable Resources

**DOI:** 10.1155/2023/9983499

**Published:** 2023-05-17

**Authors:** Mingshi Chen, Yang Liu, Xiaoxue Bao, Yuhua Yue, Binbin Tong, Xionghui Yang, Hui Yu, Ying Yang, Yuhong Liu, Yingying Yu

**Affiliations:** ^1^Guangdong Provincial Key Laboratory of Animal Molecular Design and Precise Breeding, School of Life Science and Engineering, Foshan University, Foshan, Guangdong 528225, China; ^2^Sinopharm Group Dezhong (Foshan) Pharmaceutical Co., Ltd., Foshan 528225, China

## Abstract

Chinese yam (*Dioscorea polystachya* Turczaninow) by-product produced in the water extraction process is commonly directly discarded resulting in a waste of resources and environmental pollution. However, the value of Chinese yam by-product which still contains effective ingredients is far from being fully realized; hence, it has the potential to be a safe and effective feed additive in aquaculture. To investigate the impacts of Chinese yam by-product on growth performance, antioxidant ability, histomorphology, and intestinal microbiota of *Micropterus salmoides*, juvenile fish (initial weight 13.16 ± 0.05 g) were fed diets supplemented with 0% (control), 0.1% (S1), 0.4% (S2), and 1.6% (S3) of Chinese yam by-product for 60 days. The results showed that no significant difference was found in weight gain, specific growth rate, and survival among all the experimental groups (*P* > 0.05). Feed conversion ratios of the S1 and S3 groups were significantly lower than those in the control group (*P* < 0.05). SOD activity of the S3 group and GSH contents of Chinese yam by-product groups were significantly higher than those in the control group (*P* < 0.05). MDA levels of the S2 and S3 groups were significantly lower than those in the control group and the S1 group (*P* < 0.05). Besides, Chinese yam by-product could protect liver and intestine health, as well as increase the abundance of beneficial bacteria and decrease the abundance of potential pathogens. This study suggests that Chinese yam by-product has the potential to be used as a functional feed additive in aquaculture, providing a reference for efficient recovery and utilization of by-products from plant sources during processing and culturing high-quality aquatic products.

## 1. Introduction

Chinese yam (*Dioscorea polystachya* Turczaninow) is the homology of medicine and food, and it is favored by people for its good flavor, rich nutrition, and excellent health function. The main nutrient composition of Chinese yam includes moisture (86.14%), ash (1.36%), total nitrogen (0.154%), reducing sugar (0.83%), mucoitin (1.47%), starch (6.57%), crude lipid (0.52%), and crude fiber (0.64%) [1]. The active functional ingredients of Chinese yam include polysaccharide, allantoin, dioscin, polyphenol, and adenosine [2]. Chinese yam by-product is mainly derived from the processing of Chinese yam, the production of light chemical products containing Chinese yam, and the decoction of Chinese yam medicine [3]. A large number of by-products from plant sources such as Chinese yam during processing are generally disposed by stacking (mainly in the form of), landfilling, and incineration; meanwhile, they are prone to decay, bacteria, and odor after long-term stacking because of their high water content, leading to severe resource waste and serious environmental pollution, further posing high risks to human health [4, 5]. Influenced by production methods and process conditions, at least 30%-70% of the active ingredients are not completely extracted and remain in by-products [6, 7]. Chinese yam by-product still contains cellulose (up to 85.48%) [8], trace element, and active substance, indicating that Chinese yam by-product has a high medicinal and commercial value for development and utilization. Consequently, how to effectively utilize Chinese yam by-product is a key problem to reduce environmental pollution and make waste profitable [9]. Chinese yam by-product supplemented in animal feed is a promising treatment method, which can reduce the waste of resources, promote the healthy development of aquaculture, and generate economic and environmental benefits due to its low cost and readily availability. The research showed that *Rehmannia glutinosa* and Chinese yam by-product could improve the growth rate of Hu sheep [10]. However, the potential usages of Chinese yam by-product as a feed additive on aquatic animals have received little attention.

Largemouth bass (*Micropterus salmoides*) is one of the most important cultured freshwater fishes in China, with an annual production of more than 0.7 million tons in 2021 [11]. However, intensive aquaculture has accelerated water quality deterioration, causing poor growth, oxidative stress, intestinal dysbacteriosis, and low immunity of fish. It is necessary to find safe and effective feed additives in high-density culture and achieve environment-friendly and healthy aquaculture. Therefore, in this study, we aimed to evaluate the effects of Chinese yam by-product supplemented in diets on growth performance, antioxidant ability, histomorphology, and intestinal microbiota of juvenile *M. salmoides*, providing a reference for efficient recovery and utilization of by-products from plant sources during processing and culturing high-quality aquatic products.

## 2. Materials and Methods

### 2.1. Experimental Diets

Chinese yam by-product produced in the water extraction process and its main active ingredients determination (Table S1 and Figure S1-2) were provided and analyzed by Guangdong Yifang Pharmaceutical Co., Ltd. (Foshan, China). Based on the concentration of Chinese yam [12] and yam extract [13] used in the previous researches, the content of effective components in Chinese yam by-product, and the nutritional requirement of juvenile *M. salmoides*, the diets of four groups were supplemented with 0% (control), 0.1% (S1), 0.4% (S2), and 1.6% (S3) of Chinese yam by-product, respectively. All feed materials were powdered and passed through a 40-mesh sieve, precisely weighted, and remixed with a blender. The 2 mm diameter pellets were extruded by a pelletizer, air-dried, and stored at −20°C until use. The chemical composition of the experimental diets was detected by Chen [14]. Crude protein was determined using the Kjeldahl method, and crude lipid was determined using the Soxhlet extraction method. Moisture was determined by drying in an oven at 105°C for constant weight. The samples were carbonized to smokeless in an electric furnace at about 100°C and then burned in a muffle furnace at 550°C for a constant weight to measure the ash content after the sample cooling. The formulation and nutritional composition of experimental diets are shown in Table 1.

### 2.2. Animals and Sampling

The experiment was conducted according to the guidelines of the animal research ethics committee of Foshan University (approval number: 2020056). Juvenile *M. salmoides* were purchased from Foshan Sanshui Baijin Aquatic Seedling Co., Ltd. (Foshan, China). After acclimation for two weeks, 480 fish of similar weight (mean initial weight: 13.16 ± 0.05 g) were randomly assigned to four groups (four replications in each group and 30 fish in each replication) in the circulating aquaculture system for 60 days. Fish were fed twice daily to visual satiation at 8 : 00 and 18 : 00. Feed intake and dead fish were recorded every day. During the experiment, water temperature, pH, dissolved oxygen, and NH_4_-N were maintained at 27-30°C, 7.7-8.2, >6.0 mg/L, and <0.1 mg/L, respectively.

At the end of the feeding trial, the total body weight of all fish in each tank was determined after fasting for 24 h. Fish were anesthetized with 10 mg/L buffered MS-222. The blood samples were collected by caudal venipuncture using 1 mL of sterile syringes and stored in a tube containing heparin sodium at 4°C for 1 h before being centrifuged (3500 rpm, 4°C). Then, the serum was collected and stored at -80°C until analyzed. Livers and intestinal contents of six fish were sampled and snap-frozen in liquid nitrogen before storage at -80°C. Two fish were used to obtain liver and intestine samples used for histomorphology.

### 2.3. Histological Observation

Fresh livers and intestines were collected, fixed in 4% paraformaldehyde, dehydrated with a graded alcohol series, embedded in paraffin, sectioned at 5 *μ*m, and stained with hematoxylin and eosin (H&E). Sections were observed with an optical microscope and photographed at 200 X. Besides, tissues were fixed in 2.5% glutaraldehyde, rinsed with PBS buffer, and embedded in resin. Thick sections were made on an ultramicrotome, stained with 2% uranyl acetate for 15 min and then lead citrate for 5 min, and examined using transmission electron microscopy (TEM, HITACHI HT7700, 120 kv).

### 2.4. Biochemistry Assay

About 0.1 g of frozen liver samples was homogenized in precooling 0.9% physiological saline at a ratio of 1 : 9 (*w*/*v*). The homogenate was then centrifuged for 15 min (3000 rpm, 4°C) and the supernatant was collected. The superoxide dismutase (SOD, WST-1 method), glutathione (GSH, microplate test), malondialdehyde (MDA, thiobarbituric acid method), and total protein (TP, Bradford method) of the liver by methods of Wen et al. [15] and Chen et al. [16], as well as alanine aminotransferase (ALT, microplate test) and aspartate aminotransferase (AST, microplate test) of serum by the method of Reitman and Frankel [17], were determined following respective kit manufacturer guidelines (Nanjing Jiancheng Bioeng. Inst., China).

### 2.5. Intestinal Microbiome Analysis

The genomic DNA of the intestinal content samples was extracted using the QIAamp Fast DNA Stool Mini Kit (Qiagen) according to the manufacturer's instructions. PCR amplification was done using 16S rRNA region V3–V4-specific primers 341F (5′-CCTACGGGNGGCWGCAG-3′) and 806R (5′-GGACTACHVGGGTATCTAAT-3′). The amplified products were extracted, purified, and quantified using QuantiFluor™. The sequencing library was constructed and sequenced on the Illumina PE250 platform by Genedenovo Biotechnology Co., Ltd. (Guangzhou, China). Bioinformatic analyses were done using Omicsmart.

### 2.6. Statistical Analyses

Analysis results were presented as means ± standard error of the means (SEM). All data analyses were done on SPSS 21.0 (Chicago, IL, USA) and analyzed using one-way ANOVA. Where significant differences (*P* ≤ 0.05) emerged after one-way ANOVA analysis, group means were compared further using Duncan's multiple range test. If the variance of the data was uneven, Tamhane's test would be applied followed by pairwise multiple comparisons. The comparison of alpha diversity indexes was calculated by the Tukey HSD test. Statistical analysis of beta diversity was conducted by the Adonis test. The differential abundance of indicator species at the genus level and intestinal microbial community was calculated by indicator analysis and LEfSe analysis, respectively. 16S rRNA functional prediction was inferred by PICRUSt2 analysis and Welch's *t*-test.

## 3. Results

### 3.1. Growth Performance

**Figure 1:**
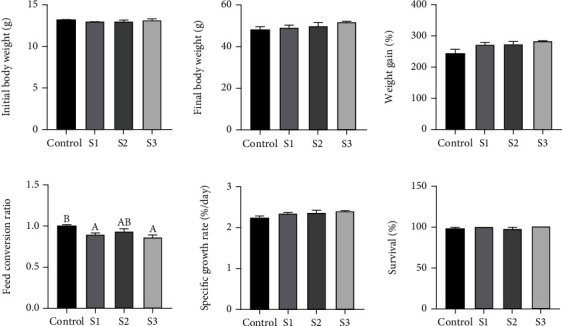
Growth performance of *M. salmoides* fed with 0% (control), 0.1% (S1), 0.4% (S2), and 1.6% (S3) Chinese yam by-product for 60 days. ^A,B^Mean values within a row without a common superscript letter were significantly different (*P* < 0.05). Data represented mean ± SEM.

The growth performance of *M. salmoides* is shown in Figure 1. Survival, weight gain (WG), and specific growth rate (SGR) did not differ significantly across experimental groups for 60 days (*P* > 0.05). However, feed conversion ratio (FCR) of the control group was significantly higher than that of the S1 and S3 groups (*P* < 0.05).

### 3.2. Biochemistry Assay

**Figure 2:**
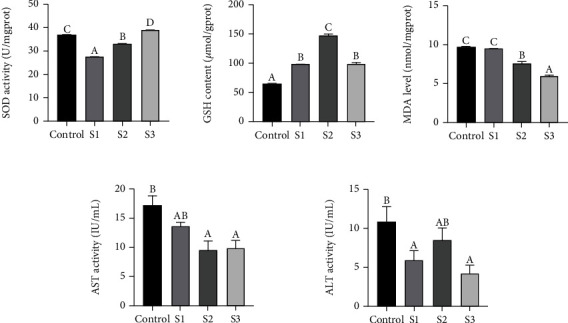
Biochemistry assay of *M. salmoides* fed with 0% (control), 0.1% (S1), 0.4% (S2), and 1.6% (S3) Chinese yam by-product for 60 days. ^A,B,C,D^Mean values within a row without a common superscript letter were significantly different (*P* < 0.05). Data represented mean ± SEM.

As shown in Figure 2, the activity of SOD of the control group was significantly higher than that of the S1 and S2 groups (*P* < 0.05), but significantly lower than that of the S3 group (*P* <0.05). The GSH content of the control group was significantly lower than that of the Chinese yam by-product groups (*P* < 0.05). MDA levels in the control and S1 groups were significantly higher than those in the S2 and S3 groups (*P* < 0.05). The AST activity of the control group was significantly higher than those in the S2 and S3 groups (*P* < 0.05); meanwhile, the ALT activity of the control group was significantly higher than that of the S1 and S3 groups (*P* < 0.05).

### 3.3. Histological Observation

**Figure 3:**
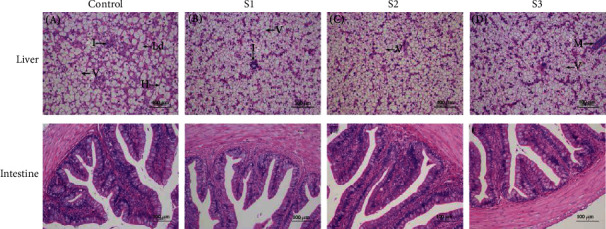
Histomorphology of liver and intestine observed in *M. salmoides* fed with 0% (control), 0.1% (S1), 0.4% (S2), and 1.6% (S3) Chinese yam by-product for 60 days. Hepatocyte hypertrophy (H), infiltration of hemocytes (I), lipid droplet (Ld), vacuolization (V), and melanism (M). Bar = 100 *μ*m, magnification ×200.

**Figure 4:**
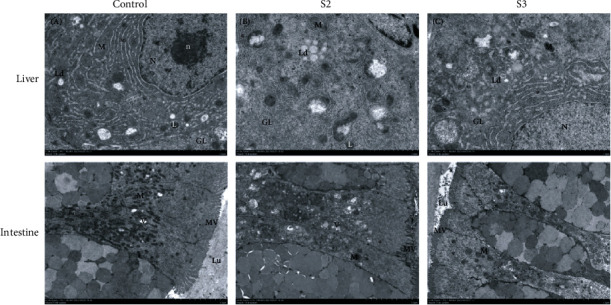
TEM micrographs of the liver and intestine of *M. salmoides* fed with 0% (control), 0.4% (S2), and 1.6% (S3) Chinese yam by-product for 60 days. ^∗^Rough endoplasmic reticulum. M: mitochondria; N: nucleus; n: nucleolus; GL: glycogen; Ld: lipid droplet; L: lysosomes, V: vacuolated; Lu: lumen; MV: microvilli. Bar = 2 *μ*m and 5 *μ*m.

Histopathological changes including infiltration of hemocytes, lipid droplets, vacuolization, and hepatocyte hypertrophy were observed in the control group (Figure 3(A)). Compared with the control, Chinese yam by-product groups had less histopathological damage (Figures 3(B)–3(D)). No histopathological abnormalities of the intestine were observed in all groups (Figures 3(A)–3(D)). Besides, TEM analysis showed that compared with the control, lipid droplet of hepatocytes and vacuolation of the intestine significantly decreased in the S3 group (Figure 4).

### 3.4. The Composition and Functional Changes of Intestinal Microbiota

**Figure 5:**
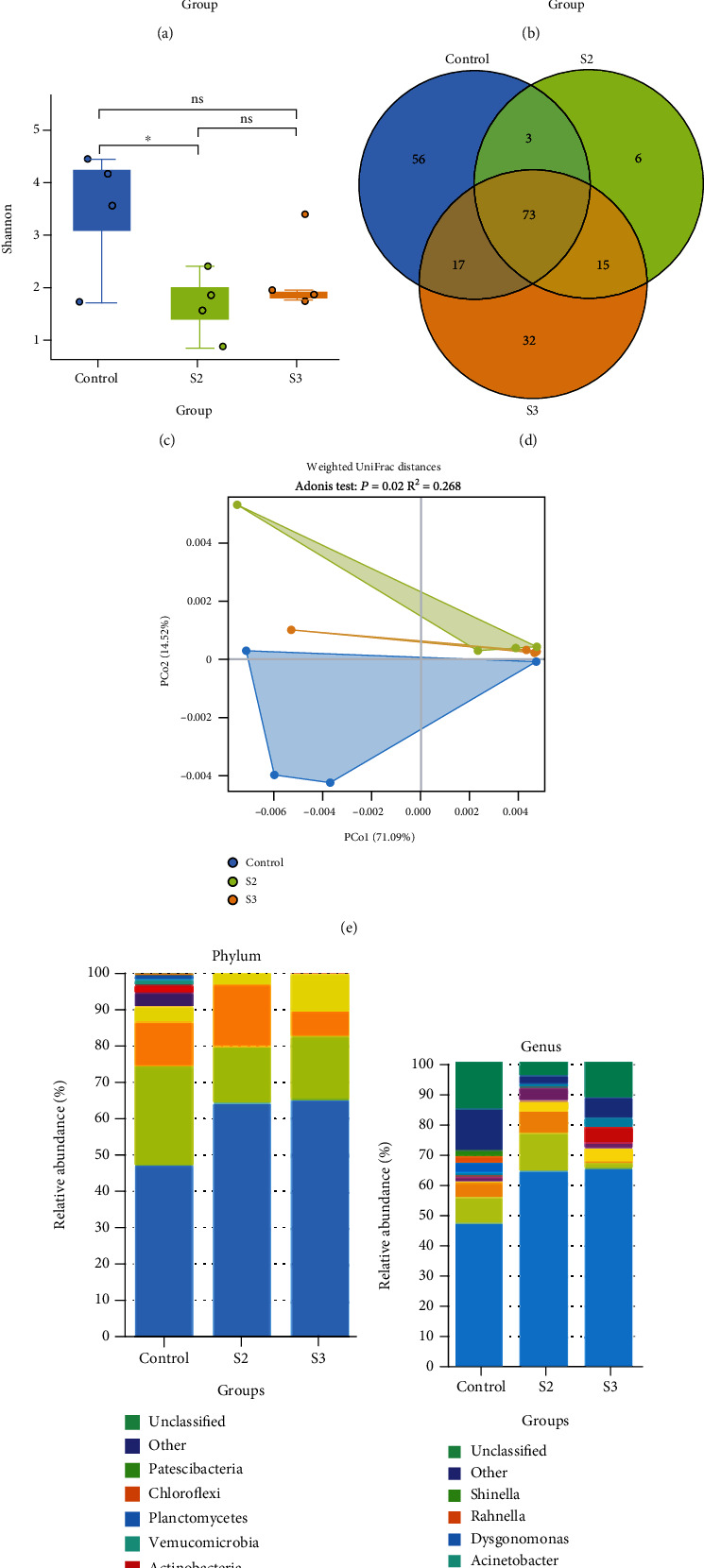
Variations of intestinal microbiota composition and comparison of the alpha and beta diversity of intestinal microbiota in the control group and the groups supplemented with Chinese yam by-product at 0.4% (S2) and 1.6% (S3) for 60 days. (a–c) Using the Tukey HSD test (ns, ^∗^, and ^∗∗^ indicated *P* > 0.05, *P* < 0.05, and *P* < 0.01, respectively). Relative proportions of the top 10 most abundant bacteria at the phylum (f) and genus (g).

As shown in Figures 5(a)–5(c), there were significant differences in Sob, Chao1, and Shannon between the control and S2 groups (*P* < 0.01, *P* < 0.05). Meanwhile, there were significant differences in Sob and Chao1 between the control and S3 groups (*P* < 0.05). Venn diagram analysis revealed that the control group, S2 group, and S3 group had 56, 6, and 32 unique genera, respectively (Figure 5(d)). The PCoA based on the weighted UniFrac distances showed that samples of the control clustered separately from those of the S2 and S3 groups (Figure 5(e)). The *P* values obtained using the Adonis test for distances were significant (*P* = 0.02). The groups supplemented with Chinese yam by-product changed the composition and diversity of intestinal microbiota at the phylum and genus levels. Tenericutes, Proteobacteria, and Firmicutes were the most abundant phyla in the control group and the S2 group, while Tenericutes, Proteobacteria, and Cyanobacteria were the most abundant phyla in the S3 group (Figure 5(f)). At the genera level, compared to the control, *Mycoplasma* in the S2 and S3 groups were significantly increased, while unclassified and other bacteria were significantly decreased (Figure 5(g)).

**Figure 6:**
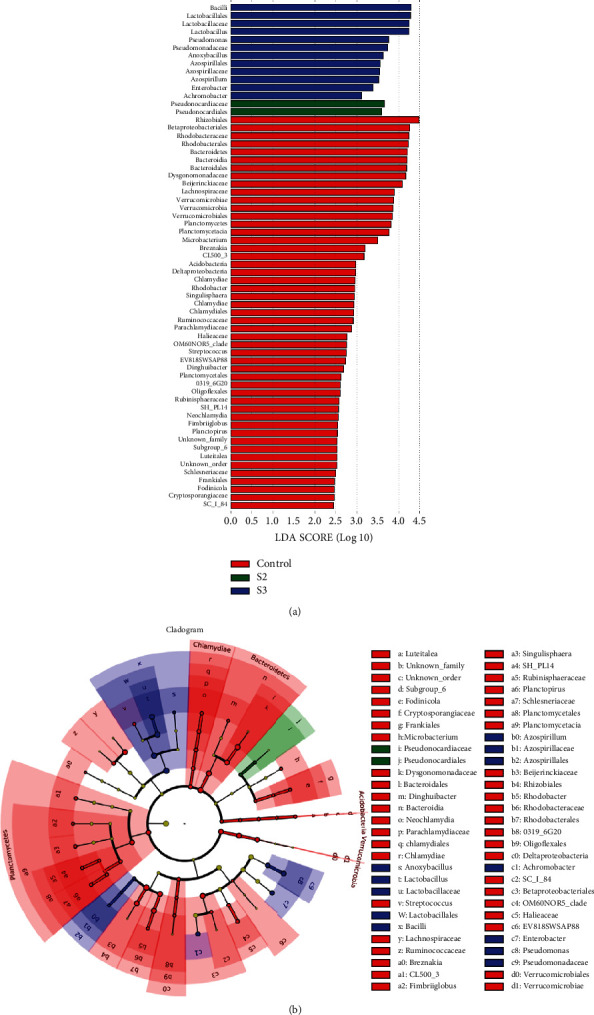
LEfSe analysis of intestinal microbial community in the control group and the groups supplemented with Chinese yam by-product at 0.4% (S2) and 1.6% (S3) for 60 days. (a) Columnar graph of LDA analysis, LDA > 2. (b) Evolutionary branch diagram.

**Figure 7:**
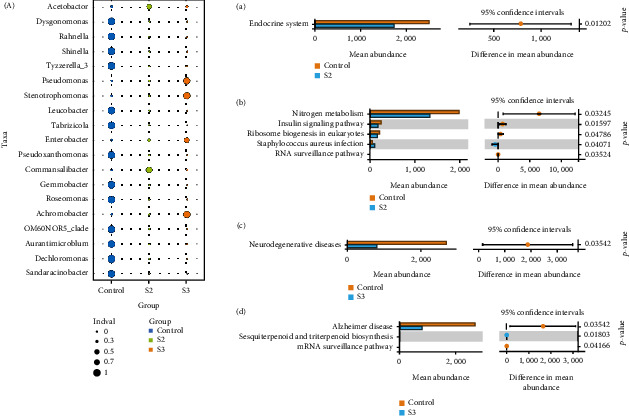
The differentially abundant taxa at the genus level (indicator analysis) and comparison in the relative abundance of PICRUSt2-generated functional profile of intestinal microbiota (a–d) in the control group and the groups supplemented with Chinese yam by-product at 0.4% (S2) and 1.6% (S3) for 60 days. The KEGG pathways were differentially abundant at level 2 (a, c) and level 3 (b, d), respectively (Welch's *t*-test).

As revealed in Figure 6, LEfSe analysis showed that *Bacilli*, *Lactobacillales*, *Lactobacillaceae*, *Lactobacillus*, *Pseudomonas*, *Pseudomonadaceae*, *Anoxybacillus*, *Azospirillales*, *Azospirillaceae*, *Azospirillum*, *Enterobacter*, and *Achromobacter* were the dominant genera in S3. *Pseudonocardiaceae* and *Pseudonocardiales* were the dominant genera in S2. Furthermore, there were 49 dominant genera in the control group, such as *Rhizobiales*, *Betaproteobacteriales*, *Rhodobacteraceae*, *Rhodobacterales*, *Bacteroidetes*, *Bacteroidia*, *Bacteroidales*, *Dysgonomonadaceae*, *Beijerinckiaceae*, *Lachnospiraceae*, and *Verrucomicrobiae*. In addition, indicator species analysis showed that compared with the control, *Acetobacter* and *Commensalibacter* in the S2 group, as well as *Pseudomonas*, *Stenotrophomonas*, *Enterobacter*, and *Achromobacter* in the S3 group increased, whereas *Dysgonomonas*, *Rahnella*, *Shinella*, *Tyzzerella 3*, *Leucobacter*, *Tabrizicola*, *Pseudoxanthomonas*, *Gemmobacter*, *Roseomonas*, OM60NOR5, *Aurantimicrobium*, *Dechloromonas*, and *Sandaracinobacter* decreased (Figure 7(A)).

The results of functional change were inferred from PICRUSt2 analysis. At KEGG level 2, compared with the control S2 and S3 groups, endocrine system and neurodegenerative diseases were enriched in the control group, respectively (Figures 7(a) and 7(c)). Functional pathways at level 3, including nitrogen metabolism, insulin signaling pathway, and ribosome biogenesis in eukaryotes were enriched in the control group (Figures 7(b) and 7(d)).

## 4. Discussion

This is the first study to evaluate the growth performance, antioxidant ability, histomorphology, and intestinal microbiota of juvenile *M. salmoides* fed with yam by-product in diets. In this study, the result showed that Chinese yam by-product did not exert a significant effect on WG, SGR, and survival. Similar with this study, after the 56-day feeding trial, Chinese yam at concentrations of 1% and 2% had no remarkable difference in WG, SGR, and survival of *Cyprinus carpio* [18]. Yam extract at concentrations of 0.1%, 0.2%, and 0.4% [13] and Chinese yam peel at concentrations of 0.5%, 1%, and 2% [19] had no significant difference in WG and SGR of fish after the 56-day feeding trial. However, after the 28-day trial, Chinese yam at concentrations of 1% and 2% [20] and *Dioscorea opposita* beans at concentrations of 3% and 5% [21] could significantly improve the growth of weaned piglets. Chinese yam at a concentration of 4% significantly improved the daily gain, feed intake, and FCR of black-bone chicken after the 8-week trial [22]. Based on the result in this study, we speculated that the low concentrations of Chinese yam by-product supplemented in diets did not exert a significant impact on the growth of juvenile *M. salmoides*. Besides, compared to livestock and poultry, the utilization of Chinese yam by fish and the environment could affect the effects of Chinese yam by-product as a feed additive on the growth of fish [23]. Besides, the results of H&E staining and TEM analysis indicated that Chinese yam by-product could reduce histopathological damage and protect the structural integrity of the liver and intestine, in agreement with previous studies [18, 24]. A study showed that compared with the control group, the groups added with concentrations of 0.5%, 1%, and 2% yam peel could increase the intestinal villus height and muscle thickness of *Carassius auratus* (*P* > 0.05) and contribute to hepatocyte integrity after an 8-week feeding trial [19]. In addition, the permeability of the cell membrane increases when the hepatocytes are damaged, and ALT and AST in the liver are released into the blood, leading to the elevation of ALT and AST levels in the serum [25]. In this study, the AST activity in the control group was significantly higher than those in the S2 and S3 groups; meanwhile, the ALT activity in the control group was significantly higher than those in the S1 and S3 groups. It further indicated that Chinese yam by-product could protect liver health.

Antioxidant defense mechanism in organisms mainly includes antioxidant enzymes and nonenzymatic complexes [26]. SOD is the most important antioxidant enzyme in eukaryotes [27]. GSH, an endogenous antioxidant and exogenous antidote, is involved in metabolic regulation [28]. MDA, one of the final products of polyunsaturated fatty acid peroxidation in the cells, is commonly known as a marker of oxidative stress and antioxidant status [29]. This study showed that the SOD activity of the S3 group and GSH contents of the Chinese yam by-product groups were significantly higher than those in the control; meanwhile, MDA levels in the control and S1 groups were significantly higher than in the S2 and S3 groups. It indicated that Chinese yam by-product had significant antioxidant stress effects. Similar with this study, previous studies have reported that Chinese yam [30] and yam polysaccharides [31] could increase SOD activity and decrease MDA level in mice. Aqueous extract of yam had obvious oxidative stress resistance in H_2_O_2_-induced *Caenorhabditis elegans* [32]. Chinese yam polysaccharides have a good antioxidant effect and can potently scavenge DPPH radical, hydroxyl radical, and superoxide radical [33]. Its hydroxyl radical scavenging activity can reach the same level of vitamin C [34]. Allantoin and phenolics from the water extract of yam peel also have antioxidative effects [35]. Therefore, we speculated that Chinese yam by-product displayed superior properties in antioxidation associated with the principal component of polysaccharide, allantoin, and phenolics.

Intestinal microbiota can prevent the colonization of infectious agents, enhance the host mucosal immunity and function, and promote the digestion and absorption of nutrients [36, 37]. In this study, the most abundant phyla in the control and S2 groups were Tenericutes, Proteobacteria, and Firmicutes. Similarly, Tenericutes, Proteobacteria, and Firmicutes were the dominant bacteria in *M. salmoides* in all groups after the 60-day Azomite feeding test [38], while Tenericutes, Proteobacteria, and Cyanobacteria were the top three abundant phyla in the S3 group. The results showed that Chinese yam by-product significantly affected the composition of intestinal bacteria of juvenile *M. salmoides*, and there might be a competitive relationship among the top three phyla. At the genus level, Chinese yam by-product affected the abundance of beneficial bacteria such as *Lactobacillus*, *Bacilli*, *Enterobacter*, *Acetobacter*, and *Achromobacter* and potential pathogens such as *Bacteroidetes*, *Shinella*, *Stenotrophomonas*, *Dechloromonas*, and *Rhodobacter*. For instance, *Lactobacillus*, *Enterobacter*, and *Pseudomonas* are the common probiotics used in aquaculture [39]. *Acetobacter* can produce by-products such as acetic and gluconic acids, effectively inhibiting the growth of some common pathogenic bacteria associated with food poisoning [40]. Although *Stenotrophomonas* species are considered to be opportunistic pathogens affecting human and plants, they can secrete important secondary metabolites and have a promising biocontrol efficacy in recent times [41]. *Achromobacter* can reduce oxidative stress by increasing amounts of superoxide anion and improving SOD activity [42]. A study has reported that *Spirulina platensis* polysaccharide could increase the abundance of *Achromobacter* of *Caenorhabditis elegans* to regulate SOD [43]. Thus, in this study, Chinese yam by-product at a concentration of 1.6% potentially regulated the abundance of *Achromobacter* to improve the antioxidant capacity by regulating the SOD activity. Due to its obvious inhibition of some intestinal bacteria such as potential pathogens, there was a decline in the diversity of intestinal bacteria in *M. salmoides* fed with Chinese yam by-product for 60 days as indicated by the low values of Sob, Chao1, and Shannon indexes. Although these disease-related bacteria were not necessarily pathogenic, these bacteria might suggest that the intestine of *M. salmoides* was in a potentially pathogenic environment. Therefore, Chinese yam by-product may have a positive impact on reducing the number of pathogenic bacteria and increasing the number of beneficial bacteria to enhance the homeostasis of the intestinal microbiota. Remarkably, previous studies reported that Chinese yam significantly improved the diversity of intestinal flora in mice [44, 45]. Diet supplemented with concentrations of 1% and 2% Chinese yam [18] and yam peel [23] changed the microbial community characteristics and markedly increased Shannon and Simpson of *Cyprinus carpio* after an 8-week feeding trial. These studies were inconsistent with the present study. A possible reason for this finding was that the experimental species are different. Further research is needed to investigate the exact mechanism of Chinese yam by-product in *M. salmoides*.

## 5. Conclusion

Overall, the present study demonstrated that the addition of Chinese yam by-product to the diet of *M. salmoides* could result in significant improvements in antioxidant capacity, liver and intestine health, and intestinal flora after a 60-day feeding trial. These findings suggest that Chinese yam by-product has the potential to be served as a valuable functional feed additive in aquaculture, allowing for the conversion of waste into a valuable resource.

## Figures and Tables

**Table 1:** Formulation and nutritional composition of experimental diets (% dry matter).

Ingredients	Control	S1	S2	S3
Fish meal	40	40	40	40
Soybean meal	20	20	20	20
Peanut meal	10	10	10	10
Wheat flour	12	11.9	11.6	10.4
Vital wheat gluten	7	7	7	7
Beer yeast	3	3	3	3
Fish oil	3	3	3	3
Soya lecithin	1	1	1	1
Choline chloride (50%)	0.5	0.5	0.5	0.5
Monocalcium phosphate	1.5	1.5	1.5	1.5
Vitamin mixture A^a^	1	1	1	1
Mineral mixture B^b^	1	1	1	1
Chinese yam by-product	0	0.1	0.4	1.6
Proximate composition				
Moisture	9.07	9.58	9.88	9.01
Crude protein	27.19	27.29	26.93	26.48
Crude lipid	11.75	11.49	10.05	12.68
Ash	12.57	12.28	12.62	12.41

^a^Vitamin mixture A (IU or mg/kg diet): VA 250000 IU, VD3 45000 IU, VC 7000 mg, riboflavin 750 mg, cyanocobalamin 1 mg, thiamine 250 mg, pyridoxine hydrochloride 400 mg, menadione 250 mg, folic acid 125 mg, biotin 10 mg, alpha-tocopherol 2.5 g, inositol 8000 mg, calcium pantothenate 1250 mg, niacin 2000 mg, and choline chloride 8000 mg. ^b^Mineral mixture B (mg/kg diet): NaCl 2.6 g, KCl 5.3 g, CaCO_3_ 37.9 g, KI 0.04 g, CuSO_4_·5H_2_O 0.02 g, ZnSO_4_·7H_2_O 0.04 g, CoSO_4_·7H_2_O 0.02 g, FeSO_4_·7H_2_O 0.9 g, MnSO_4_·H_2_O 0.03 g, CaHPO_4_·2H_2_O 9.8 g, and MgSO_4_·7H_2_O 3.5 g.

## Data Availability

The data used to support the findings of this study are included within the article and the supplemental file.

## Supplementary Materials

Supplementary Materials Table S1: the content of adenosine and allantoin in Chinese yam by-product. Figure S1: chromatogram of adenosine in Chinese yam by-product and its standard. Figure S2: chromatogram of allantoin in Chinese yam by-product and its standard.
