# Supplementary material for: Potential of Chinese Yam (Dioscorea polystachya Turczaninow) By-Product as a Feed Additive in Largemouth Bass (Micropterus salmoides): Turning Waste into Valuable Resources
Source: Aquac Nutr. 2023 May 17;2023:9983499. doi: 10.1155/2023/9983499 (PMC10208758; doi:10.1155/2023/9983499)
Supplement: Supplementary Materials — Table S1: the content of adenosine and allantoin in Chinese yam by-product. Figure S1: chromatogram of adenosine in Chinese yam by-product and its standard. Figure S2: chromatogram of allantoin in Chinese yam by-product and its standard. [file 9983499.f1.docx]

Table S1 The content of adenosine and allantoin in Chinese yam by-product.

| Sample | | Peak area | Concentration (μg/mL) | Content (mg/g) | Percentage (%) |
| --- | --- | --- | --- | --- | --- |
| Adenosine | Standard | 80.177 | 5.3614 | / | / |
|  | Chinese yam by-product | 19.554 | 1.3076 | 0.1080 | 0.01 |
| Allantoin | Standard | 253.081 | 147.2000 | / | / |
|  | Chinese yam by-product | 36.039 | 20.9614 | 1.7387 | 0.17 |

Fig. S1 Chromatogram of adenosine in Chinese yam by-product and its standard.

Fig. S2 Chromatogram of allantoin in Chinese yam by-product and its standard.
